# Grading of Age-Related Macular Degeneration: Comparison between Color Fundus Photography, Fluorescein Angiography, and Spectral Domain Optical Coherence Tomography

**DOI:** 10.1155/2013/385915

**Published:** 2013-05-08

**Authors:** Nils F. Mokwa, Tina Ristau, Pearse A. Keane, Bernd Kirchhof, Srinivas R. Sadda, Sandra Liakopoulos

**Affiliations:** ^1^Cologne Image Reading Center and Laboratory, Department of Ophthalmology, University of Cologne, 50924 Cologne, Germany; ^2^Biomedical Research Centre for Ophthalmology, Moorfields Eye Hospital NHS Foundation Trust and UCL Institute of Ophthalmology, London EC1V 2PD, UK; ^3^Doheny Image Reading Center, Doheny Eye Institute, Keck School of Medicine of the University of Southern California, Los Angeles, CA 90033, USA

## Abstract

*Purpose*. To compare color fundus photography (FP), fluorescein angiography (FA), and spectral domain optical coherence tomography (SDOCT) for the detection of age-related macular degeneration (AMD), choroidal neovascularisation (CNV), and CNV activity. *Methods*. FPs, FAs, and SDOCT volume scans from 120 eyes of 66 AMD and control patients were randomly collected. Control eyes were required to show no AMD, but other retinal pathology was allowed. The presence of drusen, pigmentary changes, CNV, and signs for CNV activity was independently analyzed for all imaging modalities. *Results*. AMD was diagnosed based on FP in 75 eyes. SDOCT and FA showed sensitivity (specificity) of 89% (76%) and 92% (82%), respectively. CNV was present on FA in 68 eyes. Sensitivity (specificity) was 78% (100%) for FP and 94% (98%) for SDOCT. CNV activity was detected by SDOCT or FA in 60 eyes with an agreement in 46 eyes. Sensitivity was 88% for SDOCT and 88% for FA. FP showed sensitivity of 38% and specificity of 98%. *Conclusions*. CNV lesions and activity may be missed by FP alone, but FP may help identifying drusen and pigmentary changes. SDOCT is highly sensitive for the detection of AMD, CNV, and CNV activity; however, it cannot fully replace FA.

## 1. Introduction

Prior to the antivascular endothelial growth factor (anti-VEGF) era, age-related macular degeneration (AMD) was considered the leading cause of severe visual loss and blindness in the developed world among people over the age of 50 years [1]. Various imaging methods are available for the diagnosis and classification of AMD. Until recently, color fundus photography (FP) was the gold standard for grading and staging in AMD clinical trials [2–4]. By permitting visualisation of the choroidal and retinal microcirculation and providing detailed information about the presence of pathological vessels as well as the integrity of the blood retinal barrier, fluorescein angiography (FA) had become a central tool for detecting and classifying CNV as well as CNV activity in eyes with neovascular AMD [5, 6].

During the past years, OCT has dramatically gained importance for the diagnosis and management of patients with chorioretinal disease by noninvasively providing cross-sectional images of the neurosensory retina and the subretinal space, thus allowing a detailed characterization of structural changes. Thus, OCT is increasingly used to determine the presence and activity of CNV and the need for (re-) treatment [7–9]. New-generation spectral domain OCT (SDOCT) instruments provide even higher resolution and more dense coverage of the macular area compared with time domain OCT [10]. Therefore, SDOCT imaging is now widely used for the followup of patients with CNV undergoing anti-VEGF therapy. 

This study aims to compare FP, FA, and SDOCT imaging regarding their sensitivity and specificity for detecting AMD, CNV, and CNV activity and to analyze whether SDOCT may have the potential to replace the other imaging techniques. 

## 2. Materials and Methods

### 2.1. Data Collection

The European Genetic Database (EUGENDA), a database collecting AMD patients as well as healthy controls, was retrospectively reviewed, and FP, FA, and SDOCT images of 120 eyes of 66 consecutive patients were randomly collected. Eyes with early, intermediate, or late AMD as well as control cases were included. Control eyes were required to show no signs for AMD, but other chorioretinal diseases including CNV secondary to any other disease but AMD was allowed. To be eligible for this study, all images had to be performed on the same day at the University of Cologne, Germany.

FPs were performed using the Canon 60 UVi fundus camera. For all patients, one 40° stereo pair centred on the fovea was captured. FA images were performed using the Spectralis HRA system (Heidelberg Engineering, Heidelberg, Germany). The standard protocol included 30° stereo images of the transit phase, mid phase, and late phase up to 10 minutes following intravenous injection of fluorescein. SDOCT images were acquired using the Spectralis SDOCT instrument (Heidelberg Engineering, Heidelberg, Germany). SDOCT volume scans (15° × 20°) composing of 37 parallel OCT B-scans were used for analysis. For each OCT B-scan, 20 images were averaged using the automated real-time (ART) function. 

### 2.2. Image Analysis

Images were independently analyzed by reading center graders (TR, NFM, and SL) at the Cologne Image Reading Center (CIRCL), which have been trained and certified in image interpretation of AMD patients. Discrepancies between graders have been solved by open adjudication. 

**Figure 1:**
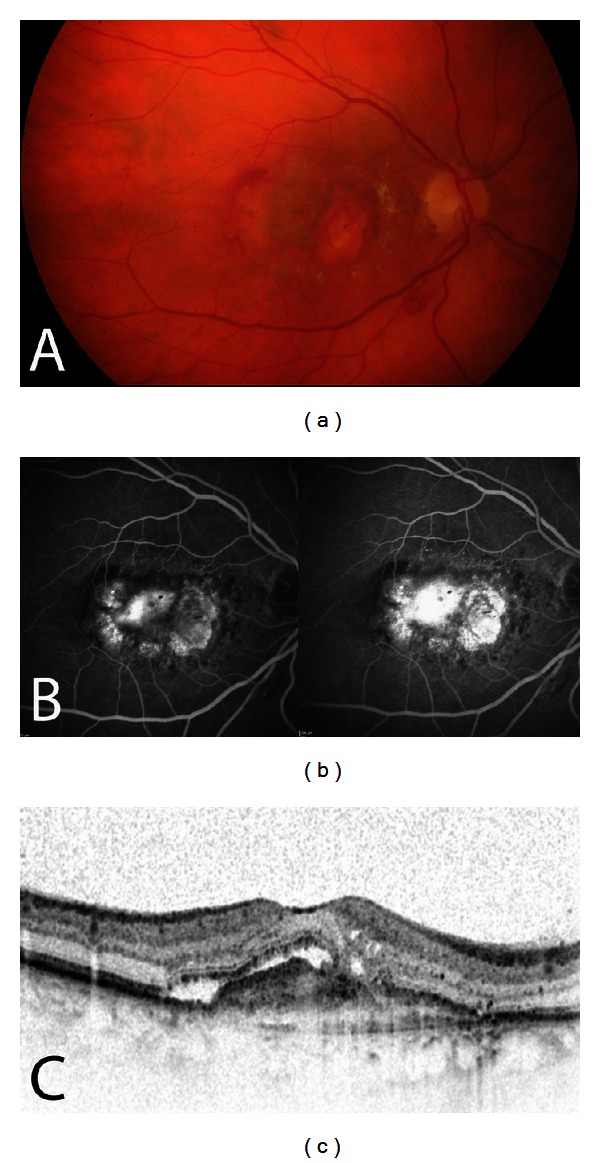
Eye with active CNV. Color fundus photography (a) demonstrates hemorrhage and fibrovascular tissue, fluorescein angiography (b) shows classic and occult CNV leakage, and SDOCT (c) presents intraretinal cystoid spaces, subretinal fluid, subretinal hyperreflective material, and a pigment epithelial detachment.

 During analysis of one imaging technique, the grader was masked to all other images and grading results of the patient. For all images, the presence of AMD, CNV, and CNV activity was noted (Table 1, Figure 1).

AMD was defined as the presence of ≥10 small (≤63 *μ*m), hard drusen and pigmentary changes or at least 1 intermediate (64–124 *μ*m) or large (≥125 *μ*m) drusen inside the 6 mm ETDRS grid.

CNV was considered present on FP, if subretinal or subRPE fibrosis and fibrovascular tissue or fibrin were seen; on SDOCT, subretinal hyperreflective material or pigment epithelial detachments (PEDs) other than single drusen were considered signs for CNV. On FA, CNV lesions were graded according to the modified Macular Photocoagulation Study (MPS) grading protocol utilized in the treatment of AMD with photodynamic therapy (TAP) and verteporfin in photodynamic therapy (VIP) studies [11, 12]. Briefly, classic CNV was identified as an area of uniform early hyperfluorescence that showed extensive leakage in the mid and late phases. Occult CNV was classified as areas of stippled hyperfluorescence that appeared in the mid and late phases of the fluorescein angiography. CNV was graded as present on FA, if classic or occult CNV lesion components or staining scar tissue was detected.

CNV activity was noted, if fluid or hemorrhage was present on FP that was not related to any other retinal vascular disease but CNV, if classic or occult CNV leakage was detected on FA or if diffuse or cystoid intraretinal fluid or subretinal fluid accumulation was seen on SDOCT. 

### 2.3. Statistical Methods

For each parameter to be evaluated, the following imaging modalities were defined as the gold standard: for presence of AMD, FP was used as the gold standard. For the presence of CNV, FA was defined as the gold standard. CNV activity was considered present if it was detected on either SDOCT or FA (ground truth). Sensitivity and specificity values for each imaging modality were calculated against the gold standard. This study adhered to the tenets set forth in the Declaration of Helsinki.

## 3. Results

A summary of grading results is provided in Table 2.

Seventy-five eyes were diagnosed with AMD based on FP. Signs for AMD were detected on FA in 77 eyes with a sensitivity of 92% (69 out of 75) and a specificity of 82% (in 8 cases, AMD was noted on FA but not on color fundus photographs). Disagreement between FA and FP was mainly related to small drusen that have been noted on FA but not on FP, and RPE hyperpigmentation that has been seen on FPs but not on FAs. On SDOCT, AMD was considered present in 78 eyes with a sensitivity of 89% (67 out of 75) and a specificity of 76% (in 11 cases, signs for AMD were noted on SDOCT but not on FP). Disagreement between SDOCT and FP could mainly be explained by small or intermediate drusen and RPE changes that have been missed on SDOCT and by intermediate drusen that have been noted on SDOCT but not on FPs.

Forty-five eyes were included in the control group. Twenty out of those eyes showed pathologies other than AMD, including high myopia, chorioretinitis, retinal vein occlusion, epiretinal membranes, diabetic retinopathy and central serous retinopathy, or idiopathic CNV. 

CNV was diagnosed in 68 eyes using FA as the gold standard. Fifty-four out of those cases were diagnosed with AMD based on FPs. In 14 cases, CNV was seen in the control group (idiopathic or related to high myopia or chorioretinitis). FP showed a sensitivity of 78% (53 out of 68 eyes) for the detection of CNV, and a specificity of 100%. SDOCT images showed signs for CNV in 64 out of the 68 cases (94%), specificity for detecting CNV was 98% (in one case, CNV was diagnosed based on SDOCT but not on FA). In the 64 cases with agreement between FA and SDOCT regarding the presence of CNV, a classic CNV lesion component was detected on FA in 25 eyes. SDOCT revealed subretinal hyperreflective material in all of these cases. Additionally, subretinal hyperreflective material was seen in 15 eyes without classic CNV leakage. Ten out of those 15 cases showed staining scar as a lesion component on FA, and 5 cases demonstrated occult CNV leakage only. Thirty-eight out of the 64 cases showed occult CNV lesion components on FA, with all of those demonstrating a PED on SDOCT. In addition, a PED was seen in 22 eyes without occult CNV lesion components on FA, with 18 (82%) out of those cases demonstrating staining scar as a lesion component and 4 (18%) cases showing classic CNV leakage only.

Out of the 68 cases with CNV diagnosed based on FA, a total of 60 cases (88%) showed signs for active CNV either on SDOCT (53 eyes) or FA (53 eyes), with an agreement between both imaging modalities in 79% out of all 68 cases (46 cases showed active CNV and 8 eyes no signs for CNV activity on both imaging modalities). In 7 cases, fluid was detected on SDOCT without evidence for CNV leakage on FA and vice versa. If the ground truth for SDOCT and FA was considered the gold standard for CNV activity, sensitivity was 88% for FA, 88% for SDOCT, and 38% (23 out of 60) for FP, respectively. Specificity for FP was 98% (in one case, CNV activity was suspected based on FP but not seen on FA or SDOCT).

## 4. Discussion

SDOCT is increasingly used in clinical trials as well as in clinical practice for the diagnosis and followup of patients with neovascular AMD undergoing anti-VEGF therapy [13]. As a noninvasive imaging tool, it provides high-resolution cross-sectional images of retinal pathology, allowing to qualitatively and quantitatively analyze various parameters relevant for (re-) treatment decisions. Our study confirms that SDOCT is highly sensitive for detecting AMD, CNV, and CNV activity; however, it may not yet fully replace the information provided by FA and FP. 

The presence of characteristic features of AMD on FP such as drusen and RPE changes was missed on SDOCT in 11% of cases in our study. This may be explained by the SDOCT volume scan settings used, as the gap between two parallel OCT B-scans was approximately 120 *μ*m; thus pathological changes may fall in between two adjacent scans and may be overlooked or may appear smaller than they truly are. On the other hand, AMD was diagnosed on SDOCT based on the presence of intermediate drusen in 24% of cases that were graded as control cases on FP. On FP, those pathological features have been either interpreted as RPE changes or small drusen, or they have been overlooked due to reduced image clarity. Thus, SDOCT may be helpful in identifying drusen and in differentiating drusen from hypopigmentation and thus may improve the quality of image interpretation in eyes with AMD compared to FP imaging alone. However, care should be taken not to transfer size definitions for intermediate or large drusen from FPs to SDOCT, as due to their shape, drusen may appear larger on SDOCT compared to FP, or they may appear smaller if they are captured at the border [14]. Further studies are needed to compare drusen sizes between those different imaging modalities before SDOCT imaging can be reliably used for staging of early and intermediate AMD in clinical trials. Other imaging techniques such as autofluorescence imaging provide additional information concerning drusen and RPE changes and may thus be helpful to identify and classify those features.

FA is commonly used as the gold standard for evaluating CNV lesions. Based on FA, CNV lesions components are categorized as classic or occult CNV leakage or staining scar tissue that may develop over time and indicate longstanding disease with poor visual function [11]. SDOCT appeared to be highly sensitive and specific in detecting CNV in our study. Do et al. reported a sensitivity of only 40% for the detection of new-onset CNV on time-domain OCT [15]. The low sensitivity may be explained by the use of time-domain OCT, as pathological features may be overlooked more easily compared to SDOCT due to the less dense scan pattern, lower image resolution, and higher rate of movement artifacts [10].

 Occult CNV on FA is believed to correspond histologically to type 1 CNV, located between the RPE and Bruch's membrane [16]. In accordance with this, all eyes with occult CNV on FA demonstrated a PED on SDOCT in our study. In contrast, classic CNV lesion components on FA histologically correspond to type 2 CNV, positioned in the subretinal space [16]. Thus, type 2 CNV lesion components are expected to present as hyperreflective material in the subretinal space on OCT. This could be confirmed in our study as all cases with classic CNV lesion components on FA demonstrated subretinal hyperreflective material on SDOCT. However, in order to correctly interpret OCT images, it is crucial to consider that OCT scans only represent “pseudohistological” images, created using information about the reflectivity and axial distribution of various structures. Hence, subretinal hyperreflective material on OCT scans may not only represent type 2 CNV, but may include, for example, subretinal hemorrhage, fibrinous material, or photoreceptor debris. This may explain why subretinal hyperreflective material was seen in our study on SDOCT in 5 cases demonstrating occult CNV on FA without the presence of a classic CNV leakage or staining scar as lesion components.

Additionally, a PED was seen on SDOCT in 22 eyes without the presence of occult CNV on FA. In those cases, other CNV lesion components such as staining scar or classic CNV may have covered the CNV membrane located in the sub-RPE space; thus occult CNV leakage was not detectable on FA. This finding indicates that high-resolution cross-sectional images provided by SDOCT may add important information regarding subretinal and sub-RPE pathology compared to the two-dimensional en-face view of FA and FP imaging.

Agreement between SDOCT and FA regarding the activity of CNV lesions in our study was seen in 79% of all 68 cases diagnosed with CNV on FA. Seven eyes demonstrated CNV leakage on FA in the absence of intra- or subretinal fluid on SDOCT, and 7 eyes showed signs for CNV activity on SDOCT without evidence of CNV leakage on FA. This discrepancy was also described in other reports [17–19]. Khurana et al. reported a sensitivity of 90% and specificity of 47% for SDOCT to detect CNV activity seen on FA [17].

**Figure 2:**
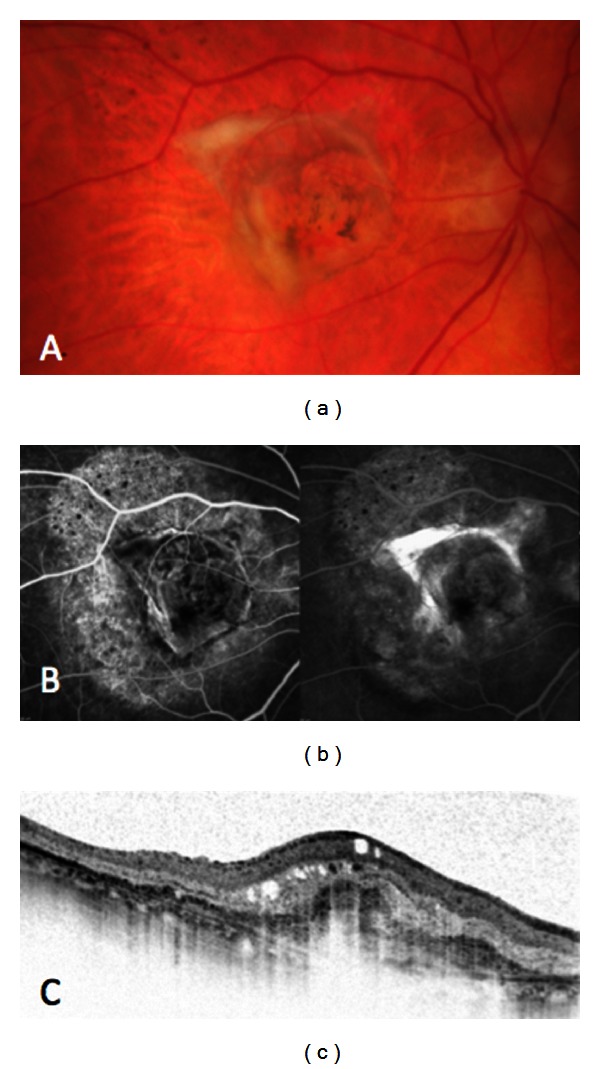
Discrepancy between imaging modalities regarding identification of signs considered to indicate CNV activity: color fundus photography (a) demonstrates fibrosis and RPE changes, fluorescein angiography (b) demonstrates staining of CNV, and SDOCT (c) shows intraretinal cystoid spaces.

The disagreement between both imaging modalities may be explained by the fact that FA and SDOCT imaging provides different information about retinal pathology. FA is used to obtain information about the perfusion and the growth of new vessels as well as the integrity of the blood-retinal barrier; thus fluorescein leakage over time can be seen during angiography. This information is missing on OCT images; thus OCT provides detailed information about pathological changes like, for example, the presence of cystoid spaces; however, it is not possible to detect whether they are caused by fluid accumulation from acute leakage from pathological vessels. Thus, cystoid spaces on SDOCT may not necessarily correspond to fluorescein leakage on FA, but may represent structural defects indicating chronic disease (Figure 2). Increase or decrease in the amount of fluid seen on OCT may thus more reliably indicate CNV activity than the presence of fluid seen at one time point. In addition, care should be taken to not confuse intraretinal cystoid spaces or subretinal fluid with “outer retinal tubulations,” a SDOCT finding described by Zweifel et al. [20]. In their paper, the authors state that degenerating photoreceptors may become arranged in a circular or ovoid fashion in chronic diseases affecting the outer retina and RPE.

In contrast, CNV activity seen on FA may be missed on SDOCT if only intraretinal cystoid spaces and subretinal fluid accumulation are considered to represent CNV activity on SDOCT. Giani et al. recently reported that intraretinal hyperreflective flecks and the inherent reflectivity and boundary definition of subretinal hyperreflective material may indicate active CNV even in the absence of intra- or subretinal fluid accumulation [21]. Additionally, fluid accumulation in the sub-RPE space such as serous components of a PED may indicate CNV activity.

FA imaging at baseline in addition to SDOCT is helpful to assess the CNV lesion subtype and the initial severity of CNV leakage; during followup, FA may confirm evidence of CNV activity whenever SDOCT interpretation is challenging or inconsistent with retinal function.

## 5. Conclusions

SDOCT, FA, and FP imaging provide complementary information about pathological changes in chorioretinal diseases. Our study indicates that drusen and RPE changes as signs for AMD are best appreciated on FP. SDOCT is highly sensitive to identify CNV and CNV activity; however, it cannot fully replace FA in the management of patients with CNV. Further studies are needed to evaluate which SDOCT parameters (e.g., cystoid spaces, diffuse intraretinal fluid, subretinal or sub-RPE fluid, inherent boundary definition of subretinal hyperreflective material, or a change in the amount of fluid) best indicate CNV activity.

## Figures and Tables

**Table 1:** Parameters evaluated as signs for AMD, CNV, and CNV activity.

	Color fundus photography	Fluorescein angiography	SDOCT
AMD	Presence of ≥10 small hard drusen and pigmentary changes or ≥1 intermediate or large drusen inside the 6 mm ETDRS grid		

CNV	Fibrosis, fibrovascular tissue, or fibrin either subretinal or subRPE (not related to any other retinal vascular disease but CNV)	Classic or occult CNV or staining scar	Subretinal hyperreflective material or PED other than single drusen

CNV activity	Fluid or hemorrhage related to CNV	Classic or occult CNV leakage	Diffuse or cystoid intraretinal fluid or subretinal fluid

AMD: age-related macular degeneration; CNV: choroidal neovascularization; FA: fluorescein angiography; SDOCT: spectral-domain optical coherence tomography; RPE: retinal pigment epithelium; and PED: pigment epithelial detachment.

**Table 2:** Sensitivity and specificity in detecting AMD, CNV, and CNV activity.

	Color fundus photography	Fluorescein angiography	SDOCT
AMD *n* (sensitivity/specificity)	75 (gold standard)	77 (92%/82%)	78 (89%/76%)
CNV n (sensitivity/specificity)	53 (78%/100%)	68 (gold standard)	69 (94%/98%)
CNV activity n (sensitivity/specificity)	24 (38%/98%)	Ground truth used as gold standard (*n* = 60)	
		53 (sensitivity 88%)	53 (sensitivity 88%)

AMD: age-related macular degeneration; CNV: choroidal neovascularization; and SDOCT: spectral-domain optical coherence tomography.
